# Whole Genome Sequencing and Comparative Genomic Analysis of *Chlamydia gallinacea* Field Strains Isolated from Poultry in Poland

**DOI:** 10.3390/pathogens12070891

**Published:** 2023-06-29

**Authors:** Kinga Zaręba-Marchewka, Arkadiusz Bomba, Sabine Scharf, Krzysztof Niemczuk, Christiane Schnee, Monika Szymańska-Czerwińska

**Affiliations:** 1 Department of Cattle and Sheep Diseases, National Veterinary Research Institute, Al. Partyzantow 57, 24-100 Pulawy, Poland; kniem@piwet.pulawy.pl (K.N.); monika.szymanska@piwet.pulawy.pl (M.S.-C.); 2 Department of Omics Analyses, National Veterinary Research Institute, Al. Partyzantow 57, 24-100 Pulawy, Poland; arkadiusz.bomba@piwet.pulawy.pl; 3 Institute of Molecular Pathogenesis, Friedrich-Loeffler-Institut (Federal Research Institute for Animal Health), Naumburger Str. 96 a, D-07743 Jena, Germany; sabine.scharf@fli.de (S.S.); christiane.schnee@fli.de (C.S.); 4 Laboratory of Serological Diagnosis, National Veterinary Research Institute, Al. Partyzantow 57, 24-100 Pulawy, Poland

**Keywords:** *Chlamydia gallinacea*, chlamydiae, poultry, comparative genome analysis

## Abstract

*Chlamydia gallinacea* is an intracellular bacterium belonging to the *Chlamydiaceae* family. Poultry is considered to be the major reservoir of this agent, which has worldwide distribution and a particularly consistent worldwide occurrence in chicken flocks. The bacterium has been linked to respiratory disease in humans but without definitive confirmation; nevertheless, while it has not been proved to be the cause of human respiratory disease, a recent report from Italy verified its bird-to-human transmission. This aspect being significant for public health, more research is needed to gain insight into the infection biology of *C. gallinacea*. In this study, the genomes of eleven novel *C. gallinacea* field strains from different regions of Poland were analyzed comparatively. It was confirmed that *C. gallinacea* strains are closely related, with at least 99.46% sequence identity. They possess a conservative genome structure involving the plasticity zone with a complete cytotoxin, the type three secretion system, inclusion membrane proteins, polymorphic membrane proteins, *hct*A and *hct*B histone-like proteins, and the chlamydial protease-like activating factor exoenzyme, as well as plasmids. Genetic diversity seems to be restricted. However, some genetic loci, such as *omp*A and multi-locus sequence typing target genes, are diverse enough to enable high-resolution genotyping and epidemiological tracing.

## 1. Introduction

Chlamydiae are obligate intracellular bacteria belonging to the two genera, *Chlamydia* and *Chlamydiifrater*, in the family *Chlamydiaceae* [1]. The genus *Chlamydia* currently includes 15 characterized species (*C. abortus*, *C. psittaci*, *C. avium*, *C. gallinacea*, *C. buteonis*, *C. caviae*, *C. crocodili*, *C. felis*, *C. muridarum*, *C. pecorum*, *C. pneumoniae*, *C. poikilotherma*, *C. serpentis*, *C. suis*, and *C. trachomatis*) [2,3,4,5,6] and four *Candidatus* species (*Cand.* *C. ibidis*, *Cand.* *C. sanzinia*, *Cand.* *C. corallus*, and *Cand.* *C. testudinis*) [7,8,9,10]. Chlamydiae have worldwide distribution and occur in a wide range of hosts: humans, companion animals, livestock, including poultry, and wildlife and exotic species. The most common *Chlamydia* species reported in birds is *C. psittaci*. Species long known to infect birds, as well as recently described species, might also be involved in avian chlamydiosis. One emerging chlamydial agent is *C. gallinacea*, first described in 2014 by Sachse et al. [11]. *Chlamydia gallinacea* is most common in poultry, and its presence has been confirmed worldwide in flocks of chickens, turkeys, and guinea fowl including in birds in Poland [12,13,14]. Initially, it was thought that *C. gallinacea* was a commensal in the gastrointestinal tract of poultry [15]; however, subsequent studies have also proven its occurrence in cattle in China [16] and in parrots (*Eolophus roseicapillus*) in Australia [17]. Data on its pathogenicity in birds are limited. So far, in field studies on poultry, only asymptomatic shedding of *C. gallinacea* has been reported while, in experimentally infected broilers, significantly lower body weight gains [18,19] and low mortality in embryonated eggs after yolk sac inoculation with *C. gallinacea* have been observed [15,20].

Until recently, the zoonotic potential of this agent was unclear. Slaughterhouse workers in France developed atypical pneumonia after exposure to *C. gallinacea*-infected poultry, but an animal origin of the pneumonia was neither unambiguously supported nor denied [21]. However, the latest report published by Italian researchers [22] finally confirmed bird-to-human-transmission of *C. gallinacea*, based on testing sputum taken from farmers having contact with poultry. Infection was asymptomatic in both hosts, however, more investigations are needed to understand better the possible risk to public health and to design and take measures to protect poultry industry workers and minimize the risk of infection. This elucidation is crucial for human health because poultry is the meat eaten most in the world (132.3 million tons were consumed in 2021 worldwide) and is also important to Poland as a leader in poultry production in the European Union [23,24]. Taking into account the impact of *C. gallinacea* on poultry production parameters and the potential risks to human health, more investigations are needed to understand this agent more comprehensively. Molecular techniques, including whole genome sequencing (WGS), are crucial for evaluating the variability and pathogenicity of *C*. *gallinacea*. Different genetic variants of *C. gallinacea* occur in the world, and Poland also has a heterogenous *C. gallinacea* population [12,14,25]. Only four whole genome sequences of *C. gallinacea* strains were available in GenBank as of May 2022. Similar to other *Chlamydiaceae* representatives, *C. gallinacea* genomes are conserved, reduced, and possess the hallmark chlamydial virulence factors [20]. The purpose of this study was to evaluate the genetic diversity of eleven novel *C. gallinacea* field strains from different parts of Poland by comparative genomic analysis and compares the strains with reference *C. gallinacea* genomes and other *Chlamydia* spp. representatives.

## 2. Materials and Methods

### 2.1. Samples

Cloacal swabs were collected from randomly selected, apparently healthy poultry flocks in all Polish voivodeships in the course of the execution of the multiannual “Protection of animal and public health” monitoring program in the years 2019–2023. Ten random samples were collected in duplicate from each flock. Dry swabs for DNA preparation were stored at −20 °C, while swabs in *Chlamydia*-stabilizing medium for cultural isolation were stored at −80 °C. All swabs were collected by authorized veterinarians, following standard procedures and with the farmers’ consent. According to the Local Ethical Committee on Animal Testing at the University of Life Sciences in Lublin (Poland), formal ethical approval is not required for this kind of study. Detailed results of this monitoring study have not been published yet as this program is still ongoing.

**Figure 1:**
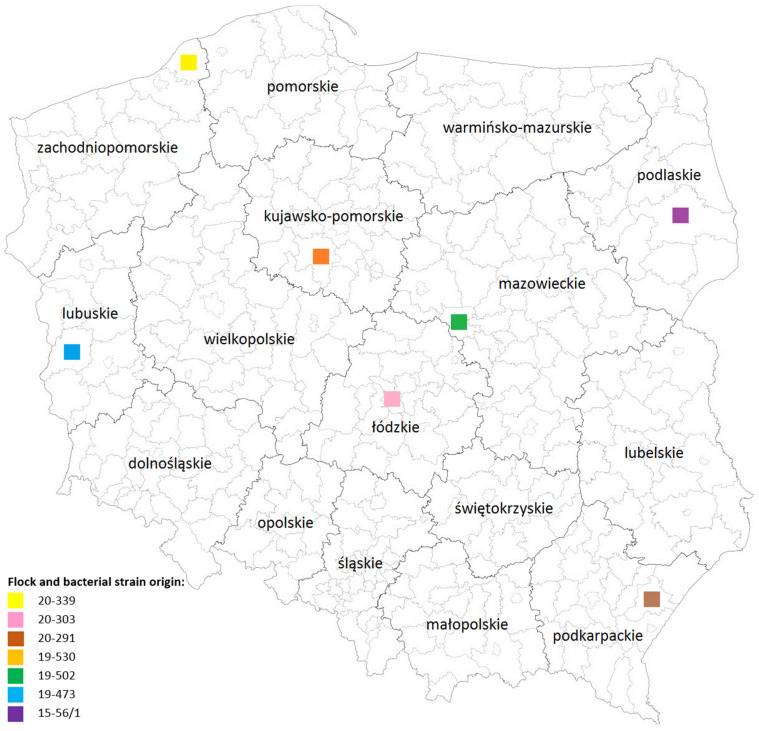
Flock (20-339, 20-303, 20-291, 19-530, 19-502, 19-473) and bacterial strain (15-56/1) origin. This map was created using Easy Software version 0.196 by Marek Roj (http://mapy.easysoftware.pl/ accessed on 2 May 2022).

Six flocks of *Gallus gallus* sampled between October 2019 and August 2020 were chosen from the positive poultry flocks and included in this survey. Each flock was located in a different voivodeship of Poland (Figure 1). Additionally, bacterial strain 15-56/1, obtained in a previous study [14], was included in this research (Figure 1).

### 2.2. DNA Extraction and Chlamydia Identification

Dry swabs were used for DNA extraction with a QIAamp DNA Mini Kit (Qiagen, Hilden, Germany) following the manufacturer’s instructions. In addition, an internal positive control (TaqMan Exogenous Internal Positive Control, Applied Biosystems, Foster City, CA, USA) was added to each sample according to the manufacturer’s protocol, in order that, in further PCR analysis, true negatives could be differentiated from negatives which were false because of inhibition. DNA extracts were stored at −20 °C before analysis. A *Chlamydiaceae*-specific real-time PCR targeting the 23S rRNA gene fragment (111 bp) [26] conserved in all members of *Chlamydiaceae* was applied for screening. Furthermore, a species-specific real-time PCR was conducted on all *Chlamydiaceae*-positive samples to identify the *Chlamydia* species, which was one of *C. gallinacea* [27], *C. psittaci* [28], *C. avium* [29], *C. abortus*, *C. pecorum*, or *C. suis* [30]. In each assay, a panel of controls was included: species-specific positive controls (*C. gallinacea*, *C. psittaci*, *C. avium*, *C. abortus*, *C. pecorum*, and *C. suis*) and negative equivalents using DNase-RNase free water (Qiagen). Threshold cycle (Ct) values above 36 were considered negative. All PCR analyses were conducted on a 7500 Real-Time PCR System (Applied Biosystems, Foster City, CA, USA).

### 2.3. Isolation and Propagation in Cell Culture

One or two cloacal swabs in stabilizing medium per flock with the highest *C. gallinacea*-DNA concentration(s) (Ct values 23.59–31.68) were chosen from six poultry flocks for isolation (Table 1) on Buffalo green monkey (BGM) cells. The isolation procedure was as described before [14] except that adjustments were made to the concentrations of nystatin (9 µg/mL), gentamycin (40 mg/mL), and vancomycin (25 mg/mL). A sample was considered positive when inclusions of typical chlamydial morphology appeared as bright apple-green spots upon staining with an IMAGEN Chlamydia kit (Oxoid Ltd., Basingstoke, UK) after two passages.

Additionally, strain 15-56/1 (Table 1) was propagated on BGM cell culture with UltraMDCK (Madin–Darby canine kidney) serum-free medium (Lonza, Cologne, Germany) in T25 flasks and incubated at 37 °C with 5% CO_2_ in a fully humidified cabinet for 48–72 h [14]. The medium was replaced after 18 h.

### 2.4. Preparation of Genomic DNA for Illumina and Nanopore (MinION) Sequencing

At 60–72 h post-infection, infected cells from four 25 cm^2^ tissue culture flasks were harvested using a scraper, frozen at −80 °C, thawed at 37 °C, and centrifuged at 25,830× *g* for 40 min at 4 °C. Supernatants were discarded and pellets were washed with ice-cold PBS, sonicated for 2 × 30 s to dissolve the pellets and centrifuged at 1000× *g* for 10 min at 4 °C to remove cellular debris. Supernatants were then centrifuged at 25,830× *g* for 40 min at 4 °C and pellets were washed with ice-cold PBS. The three-step centrifugation process was repeated. Genomic DNA was extracted from pellets with Qiagen 20/G Genomic-tips and a Genomic DNA buffer set according to the “Preparation of Gram-Negative and some Gram-Positive Bacterial Samples” protocol in the QIAGEN Genomic DNA Handbook.

DNA extracts were confirmed by analysis with a Nanodrop One spectrophotometer and Qubit 3.0 fluorometer (both from Thermo Fisher Scientific, Waltham, MA, USA) to confirm the quality and quantity. Capillary electrophoresis was applied to assess DNA integrity.

### 2.5. Illumina and Nanopore Sequencing, Assembly, and Draft Annotation

Genomic libraries were constructed using the Nextera XT DNA library preparation kit and Nextera XT index kit (Illumina, San Diego, CA, USA). Sequencing was conducted on a MiSeq sequencer (Illumina, San Diego, CA, USA) with the 2 × 300-bp paired-end protocol. For sequencing with the MinION system (Oxford Nanopore Technologies, Oxford, UK), a 1D genomic DNA sequencing protocol, in combination with an SQK-LSK109 1D ligation sequencing kit and the EXP-NBD104 and EXP-NBD114 native barcode expansion kits, were used as recommended by the manufacturer (Oxford Nanopore Technologies, Oxford, UK). Sequencing was performed in the MinION system using a FLO-MIN106D (R9.4.1) flow cell according to a standard protocol (Oxford Nanopore Technologies, Oxford, UK) and base-called with Guppy version 5.0.17. Strains 20-339/2, 20-339/3, 20-303/8, 20-291/9, 19-530/1, 19-502/5, 19-473/7, and 19-473/10 underwent this procedure. Genomic DNA of strains 20-303/10, 19-502/7, and 15-56/1 were sent for sequencing to Eurofins Genomics (Ebersberg, Germany). Illumina NovaSeq genome sequencer technology with a 2 × 150-bp paired-end protocol was applied for sequencing. The quality of the reads was checked using FastQC version 0.11.9 [31], then trimmed with fastp 0.12.4 [32], Porechop 0.2.4 (https://github.com/rrwick/Porechop accessesd on 6 April 2022) or NanoFilt 2.8.0 [33], depending on the read type. Genome sequences for the 20-339/2, 20-339/3, 20-303/8, 20-291/9, 19-530/1, 19-502/5, 19-473/7, and 19-473/10 strains were generated by combining data from both the Illumina and MinION data sets using Unicycler version 0.5.0 [34], whilst sequence reads obtained for the remaining three strains were assembled using SPAdes version 3.15.3 [35]. All genome sequences were annotated using Prokka version 1.14.5 [36]. Default parameters were used for all software programs, unless otherwise specified.

### 2.6. Phylogenetic and Genome Analysis

Pairwise similarity of genomes was found using JSpecies version 3.9.5 [37] with default parameters to determine the average nucleotide identity (ANIb) and the correlation of the tetra-nucleotide signatures between pairwise genomic comparisons of *C. gallinacea* strains and chlamydial representatives.

Whole-genome single-nucleotide polymorphism (SNP) analysis was conducted using fastq files of eleven *C. gallinacea* strains and four fasta files of reference sequences, which were input to Snippy version 4.4.3 (https://github.com/tseemann/snippy accessed on 31 January 2023). The type strain *C. gallinacea* 08-1274/3 was used as a reference genome. A phylogenic tree was built based on core SNP alignment from Snippy using RAxML-NG version 1.0.1 [38] with the substitution model GTR+G+ASC_STAM, with support values calculated from 1000 bootstraps. The phylogenetic tree was visualized with Interactive Tree of Life (ITOL) version 6.7.6 [39].

The gene sequences of a variety of important diagnostic markers and putative virulence factors such as 16S rRNA, 23S rRNA, *omp*A, the plasticity zone (PZ) fragments as well as plasmid sequences (reoriented using Circlator v.1.5.5 [40]) were extracted from whole genome sequences of *C. gallinacea* isolates and *Chlamydia* spp. by Geneious Pro 8.0 software (Biomatters, Auckland, New Zealand) or downloaded from GenBank. The acetyl-CoA carboxylase (*acc*B) and einosine-5′-monophosphate dehydrogenase (*gua*B) genes were established as the boundaries of the plasticity zone. Where the *gua*B gene was missing, the queuosine precursor transporter was substituted. The fragments of genome determined by these genes were extracted from analyzed genomes.

The multi-locus sequence typing application MLST version 2.19.0 (https://github.com/tseemann/mlst accessed on 13 June 2022) was used with default settings to query the assemblies against the PubMLST Chlamydiales database. The *gat*A, *opp*A, *hfl*X, *gid*A, *eno*A, *hem*N, and *fum*C gene sequences were extracted from *C. gallinacea* isolates. Seven house-keeping genes from 38 *C. gallinacea* and one *C. avium* reference strain (available in the PubMLST database) were included in the analysis.

Alignments were created by MAFFT [41] version 7.017 and ClustalW in Geneious Pro 8.0 [42]. Nucleotide and amino acid identities were calculated based on multiple sequence alignments in Geneious Pro 8.0. To construct dendrograms, IQ-TREE version 1.6.12 [43] with 1000 bootstrap replicates and the best-fit model according to the Bayesian information criterion were utilized, while the Model Finder tool of IQ-TREE v1.6.12 [44,45,46] was used to calculate the models. ITOL [39] was applied for the visualization of the phylogeny. Chlamydial plasmid arrangements and BLAST comparisons of plasticity zone fragments were visualized using EasyFig 2.1 [47].

The Virulence Factor Database (VFDB) (http://www.mgc.ac.cn/cgi-bin/VFs/genus.cgi?Genus=Chlamydia accessed on 20 January 2023) was used to predict chlamydial virulence genes including type III–secreted proteins. The output virulence factors were manually filtered based on datasets available in the VFDB “Effector delivery system” section for type three secretion system (T3SS) and the effectors thereof (http://www.mgc.ac.cn/cgi-bin/VFs/compvfs.cgi?Genus=Chlamydia&hl=VF0711#VF0711 accessed on 20 January 2023). The resulting hits were filtered for a bit score of minimum 50, because for average length proteins, a bit score of 50 is almost always significant [48].

Inclusion membrane proteins (Incs) and the histone-like proteins *hct*A and *hct*B were identified based on reference sequences representing different subtypes of Incs, *hct*A, and *hct*B genes available in the GenBank and UniProt databases. Their sequences were compared with sequences from the strains studied in this paper by BLASTp version 2.12.0 [49].

The DeepTMHMM version 1.0.18 tool (https://www.biolib.com/DTU/DeepTMHMM accessed on 23 November 2022) was used to predict chlamydial membrane proteins based on the presence of bi-lobed hydrophobic domains, because this tool currently provides the most complete and best-performing method for the prediction of the topology of both alpha-helical and beta-barrel transmembrane proteins [50]. The cut-off was set as 40 or more amino acids in the bi-lobed hydrophobic domain.

All sequences of polymorphic membrane proteins (pmps) of *Chlamydia* spp., available in the GenBank and UniProt databases and described by Vasilevsky [51], were used to create protein databases. All genome sequences from this study were compared with generated databases using BLASTp version 2.12.0 [49]. The resulting hits were filtered for a bit score and ‘qcovhsp’ of minimum 50 [48].

## 3. Results

### 3.1. Identification and Isolation of Chlamydia gallinacea Strains

The *Chlamydiaceae* 23S rRNA real-time PCR confirmed shedding in all six poultry flocks. *Chlamydia gallinacea* was identified in all tested poultry flocks by the species-specific real-time PCR. The presence of other *Chlamydia* species (*C. psittaci*, *C. avium*, *C. abortus*, *C. pecorum*, and *C. suis*) was excluded (Table 1). Ten novel strains were successfully isolated in total in this study: 20-339/2, 20-339/3, 20-303/8, 20-303/10, 20-291/9, 19-530/1, 19-502/5, 19-502/7, 19-473/7, and 19-473/10. The bacterial strain 15-56/1 was propagated.

### 3.2. Chlamydia gallinacea Genomes

Eleven strains were sequenced with second- and third-generation sequencing methods. All strains contained a ~1.1 Mb genome and a conserved plasmid of ~7.5 kb. For four strains (20-339/2, 20-339/3, 20-303/8, and 19-530/1), sequencing and assembly resulted in single circular chromosomal contigs while, for the remaining seven strains, multiple chromosomal contigs (*n* = 6–27) were obtained. The G+C content of the chromosomal DNA was 37.9% for all strains, whilst the G+C content of plasmidial DNA ranged from 31.6 to 31.8%. The average number of coding sequences (CDS) was about 905, which is comparable with results obtained for reference strains of *C. gallinacea*. The basic genomic parameters of these eleven strains in comparison with other *C. gallinacea* representatives are given in Supplementary Table S1. All chromosomal and plasmidial sequences, as well as the raw data obtained in this study, have been deposited in the European Nucleotide Archive (ENA) as a part of bioproject PRJEB55472 under the accession numbers shown in Supplementary Table S2.

### 3.3. Phylogenetic and Genomic Analysis

#### 3.3.1. ANIb and Tetra-Nucleotide Signatures

The ANIb was calculated by BLAST and tetra-nucleotide regressions between the sequences of the eleven Polish *C. gallinacea* field strains, four reference strains of *C. gallinacea*, and other *Chlamydia* spp. The ANIb and tetra-nucleotide regression between all *C. gallinacea* sequences ranged from 99.34% to 100% and from 0.99956 to 1, well above the respective cut off values for species separation of 95% and 0.989, and thus confirming species affiliation. In contrast, the ANIb between all analyzed *C. gallinacea* sequences, and those of other *Chlamydiaceae*, ranged between 81.17% and 67.98%. Detailed results are presented in Supplementary Table S3.

#### 3.3.2. Analysis of 16S rRNA, 23S rRNA and *omp*A

A concurring result was provided by 16S rRNA and 23S rRNA phylogenetic analysis, which assigned all analyzed strains to *C. gallinacea* with 99.9% to 100% nucleotide sequence identity at these important diagnostic marker genes.

**Figure 2:**
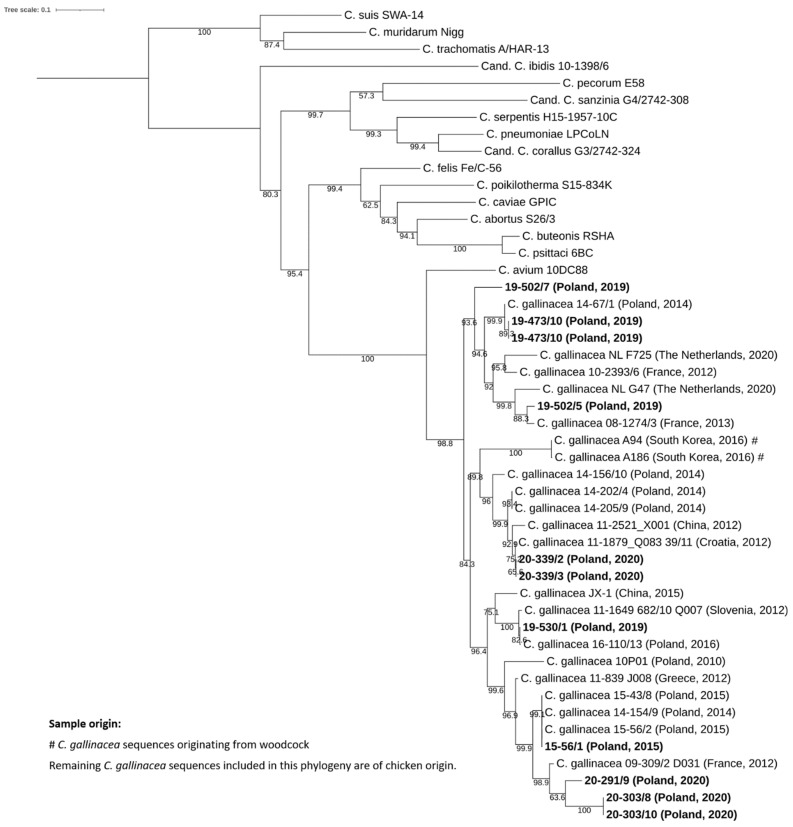
Analysis of the complete *omp*A gene of *C. gallinacea* field strains and representative strains of *Chlamydia* spp. The phylogeny based on 1266-bp consensus alignment was constructed by the maximum likelihood method with best-fit model according to the Bayesian information criterion TVM+F+I+G4. Specimens found in this study are in boldface. Bootstrap values are presented as percentages.

In contrast, *C. gallinacea omp*A sequences presented high variability with nucleotide identity ranging from 69.23% to 100% (Supplementary Table S4). The sequences from our study grouped with various reference *C. gallinacea* sequences, including European strains originating from France (08-1274/3 and 09-309/2 D031), Croatia (11-1879_Q083 39/11), Slovenia (11-1649 682/10 Q007), and Poland (14-67/1, 15-43/8, 14-154/9 and 15-56/2), but also a strain from China (11-2521_X001) (Figure 2). Therefore, *omp*A-based phylogeny did not reflect the geographical origin of *C. gallinacea* strains.

#### 3.3.3. Multi-Locus Sequence Typing

Based on the analysis of seven MLST housekeeping genes established for *Chlamydiaceae* [52] (http://pubmlst.org/chlamydiales/ accessed on 13 June 2022) (*eno*A, *fum*C, *gat*A, *gid*A, *hem*N, *hlf*X, and *opp*A), eight new sequence types (ST)—ST321–328—were determined for eleven *C. gallinacea* strains from Poland (Table 2), among which isolates from the same farm often showed the same genotype. The obtained sequences and all sequences of *C. gallinacea* representatives available in the PubMLST database (http://pubmlst.org/chlamydiales/), as well as the *C. avium* 10DC88 type strain, were included in the phylogenetic analysis, resulting in the dendrogram shown in Supplementary Figure S1. This illustrates the genetic diversity of the Polish isolates, which clustered in four distinct clades together with strains of diverse geographical origin.

#### 3.3.4. SNP-Based Analysis

**Figure 3:**
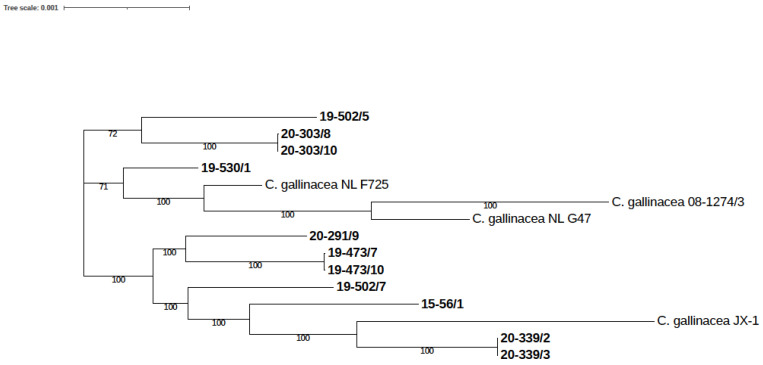
Single-nucleotide polymorphism-based phylogeny of *C. gallinacea* strains. The dendrogram was constructed based on core SNP alignment using RAxML-NG with the substitution model GTR+G+ASC_STAM, with support values calculated from 1000 bootstraps. The scale bar indicates the number of substitutions per site.

The eleven strains from this study and four *C. gallinacea* reference strains (08-1274/3, JX-1, NL F725, and NL G47) were used for whole-genome SNP analysis. A dendrogram constructed based on core SNP alignment is presented in Figure 3. The number of core SNPs between the eleven strains from this study ranged from 0 to 3613, and, between the studied strains and *C. gallinacea* reference strains, it ranged from 1611 to 4525. There were no or very few SNPs between three pairs of strains originating from the same flocks, which can be regarded as clonal: 20-339/2 and 20-339/3 (0 SNPs), 20-303/8 and 20-303/10 (5 SNPs), and 19-473/7 and 19-473/10 (6 SNPs). In contrast, strains 19-502/5 and 19-502/7 sampled from the same flock showed 2804 SNPs and clearly represent epidemiologically independent *C. gallinacea* strains (Supplementary Table S5).

#### 3.3.5. Plasticity Zone

**Figure 4:**
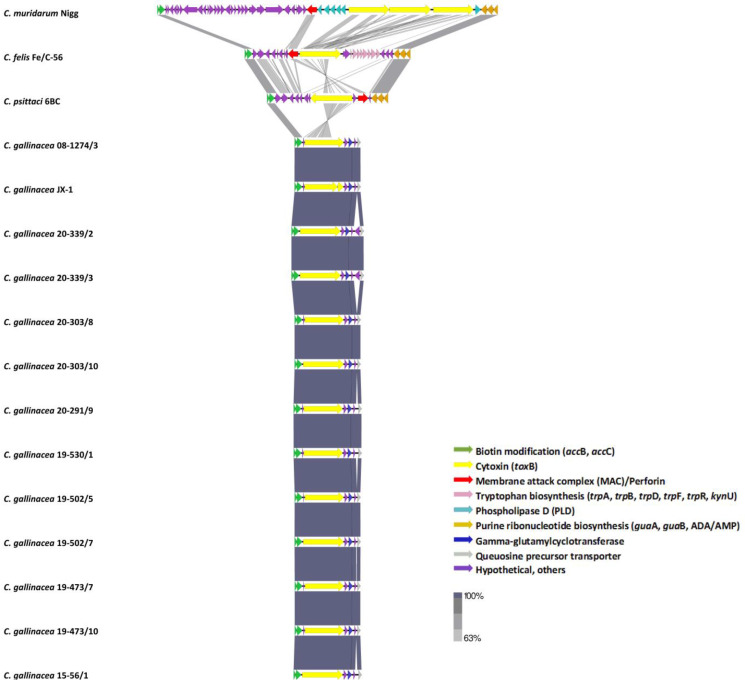
EasyFig plot of tBLASTx analysis of the plasticity zones of the eleven *C. gallinacea* strains, four *C. gallinacea* reference genomes, and three representatives of *Chlamydia* spp. Gray shading shows sequence identity, whilst colored arrows represent plasticity zone genes.

The median size of the PZ of *C. gallinacea* field strains was 15,845 bp with nucleotide identity ranging from 90.10% to 100% (Supplementary Table S6). With respect to the PZ, the novel Polish strains were closely related to the reference strains 08-1274/3 (90.89–99.80%) and JX-1 (90.62–99.14%) of *C. gallinacea*. The level of nucleotide identity was significantly lower with other avian species at 48.70–48.91% for *C. avium*, 46.67–49.13% for *Cand.* *C. ibidis*, 30.22–32.46% for *C. psittaci*, and 27.57–30.10% for *C. buteonis*. *Chlamydia gallinacea* representatives demonstrated gene content reduction in their PZs in comparison to other chlamydial species such as avian *C. abortus* 15-70d24 (20,909 bp), *C. buteonis* RSHA (26,735 bp), *C. psittaci* 6BC (29,144 bp), and *Cand.* *C. ibidis* 10-1398/6 (31,344 bp), while the PZ of *C. avium* (5694 bp) was approximately three times smaller. All PZs of *C. gallinacea* strains started with Acetyl-CoA-carboxylase (*acc*BC) and ended with the queuosine precursor transporter. Cytotoxin virulence factor (*tox*B) and gamma-glutamylcyclotransferase could be found in all *C. gallinacea* strain PZs, while purine and pyrimidine biosynthesis genes (*gua*AB-add), membrane attack complex (MAC)/perforin genes, the tryptophan operon (*trp*ABFCDR, *kyn*U, and *prs*A), and phospholipase D (PLD) were absent from this region. Interestingly, the two clonal isolates 20-339/2 and 20-339/3 had an additional CDS assigned as a hypothetical protein of unknown function and showed the lowest nucleotide identity (90.10–93.38%) with other *C. gallinacea* strains, while the remaining nine strains presented sequence homology at the level of 95.79–99.99%. Moreover, *C. gallinacea* JX-1 had a premature STOP codon in the cytotoxin virulence factor, in contrast to our *C. gallinacea* strains and other reference strains (Figure 4).

#### 3.3.6. Plasmid Comparisons

All isolated *C. gallinacea* field strains contained a plasmid of 7492–7569 bp with eight CDS, just as most *Chlamydia* spp. are known to have (Supplementary Figure S2) [53]. In all strains, integrase (CDS 1/PGP 7 and CDS 2/PGP 8), replicative DNA helicase (CDS 3/PGP 1), virulence plasmid proteins (CDS 4/PGP 2, CDS 5/PGP 3, and CDS 6/PGP 4), plasmid partitioning protein *Par*A (CDS 7/PGP 5), and plasmid replication protein (CDS 8/PGP 6) were annotated, which is congruent with results described previously for *C. gallinacea* plasmids [25]. Phylogenetic analysis of eleven plasmids from this study with various chlamydial plasmids representing other avian origin species (*C. gallinacea*, *C. avium*, *C. psittaci*, and avian *C. abortus*) showed that they cluster with *C. gallinacea* representatives and *C. avium*. The plasmid sequence similarity among *C. gallinacea* strains was high and ranged from 97.25% to 100%, while the similarity of *C. gallinacea* strains to *C. avium*, being the closest related species, was notably lower (75.96–76.78%). Detailed parameters of nucleotide identity are shown in Supplementary Table S7.

#### 3.3.7. Putative Virulence Factors and Effector Delivery System

The VFDB identified 207–215 putative virulence genes in the eleven Polish *C. gallinacea* strains. The distribution of virulence genes seems to be the same with respect to gene presence and localization. The T3SS discovered in all strains is composed of 75 structural, chaperone and secreted effector proteins, including 51 effectors. Detailed data regarding the predicted virulence genes are shown in Supplementary Table S8. The two T3SS effectors, termed translocated actin-recruiting phosphoprotein (TARP) and secreted inner nuclear membrane-associated *Chlamydia* protein (SINC), as well as the exoenzyme chlamydial protease-like activating factor (CPAF), all known for their roles in chlamydial pathogenesis, were found in all eleven strains. The phylogenetic analysis of the *tar*P and *sin*C genes found in strains from this study and *C. gallinacea* reference strains revealed that these genes are highly conserved (Supplementary Tables S9 and S10). The size of SINC orthologs in all *C. gallinacea* strains was smaller (372–373 bp) than those identified in *C. psittaci* strains (>500 bp).

Some genes which were not designated as virulence factors were checked manually by BLAST. The histone-like protein *hct*A and *hct*B genes were found with lengths of 118 aa and 187 aa, respectively, in all strains from this study. Compared to the *C. gallinacea* 08-1274/3 type strain, Polish strains revealed a high level of sequence identity with a maximum value of 100% for *hct*A and 98.39–100% for *hct*B.

#### 3.3.8. Inclusion Membrane Proteins and Membrane-Associated Proteins

Four subtypes of Inc were identified in the eleven Polish *C. gallinacea* strains as well as in the *C. gallinacea* reference strains: A (two proteins), B (one protein), C (one protein), and V (one protein). All these genes shared a high level of amino acid identity: 98.90–100% (*Inc*A), 97.85–100% (*Inc*A 2), 98.54–100% (*Inc*B), 98.78–100% (*Inc*C), and 98.62–100% (*Inc*V). The most conserved was *Inc*C with 100% similarity between all strains except strain 19-473/7, which had a truncated gene (Supplementary Table S11).

Taking into consideration the presence of bi-lobed hydrophobic domains as a common feature of membrane-bound proteins, all eleven *C. gallinacea* strains were predicted to encode from 57 to 61 putative membrane proteins. Depending on the strain, 20–25 potential membrane proteins with two transmembrane domains (including two *Inc*A and *Inc*C proteins), 16 with four transmembrane domains (including *Inc*V protein) and 20–21 with six transmembrane domains were predicted. According to the latest version of DeepTMHMM Server, which was version 1.0.18, there are no transmembrane domains in *Inc*B.

#### 3.3.9. The Family of Polymorphic Membrane Proteins

To explore the repertoire of pmps in the Polish strains, all genome sequences from this study were compared with records in a generated pmps database. Almost all results were confirmed by hits in the VFDB database (see Supplementary Table S12).

All subtypes of *pmp* genes (A, B, D, E/F, G/I, and H) were identified, which is congruent with previously published results [25,51,54]. While subtypes A, B, D, H, G1, and G2 genes were present and complete in all eleven strains, genes of the group E/F and also many of group G/I occurred only in some strains and often possessed premature STOP codons caused by frameshifts. The *pmp*05 genes, which were detected in our *C. gallinacea* strains, were homologs of *pmp*G based on comparisons with sequences from the literature [25,51,54]. The *pmp* genes, which were present and complete in all eleven Polish strains and the type strain, are conservative, with high levels of amino acid sequence identity values: 98.71–100% (A), 90.39–100% (B), 90.93–100% (D), 96.76–100% (H), 97.85–100% (G1), and 88.34–100% (G2). The one exception was strain 19-502/5 with 76.27–82.33% sequence identity.

## 4. Discussion

Identifying novel infectious diseases and predicting the locations of their next loci and the likelihood of their epidemics or crossing of species barriers are becoming possible as new technologies are constantly being implemented and improved. The recent developments in next-generation sequencing make WGS affordable, which renders deeper exploration of the biology of bacteria and viruses achievable and offers insights into pathogen diversity. Chlamydial genomics has become one of the principal pillars of molecular as well as clinical research on *Chlamydia* spp. during the last two decades [55].

*Chlamydia gallinacea* is an emerging agent which, worldwide, occurs mainly in poultry, this also being the case in Poland. Despite its high prevalence, there are only four *C. gallinacea* genomes available in GenBank: strain 08-1274/3 is the type strain originating from France (2013), strain JX-1 was isolated in China in 2015, and two Dutch strains—NL F725 and NL G47—are logged which were isolated in 2020. In this research, we presented detailed a comparative genome analysis of eleven Polish field strains isolated from chickens and originating from different locations (7 out of 16 Polish voivodeships).

Genome sequencing is a rapidly evolving field which improves the understanding of pathogens’ diversity, biology, and phylogeny. There are many competing technologies of next-generation sequencing (NGS) available to *Chlamydia* researchers; all with their unique benefits and drawbacks. Short-read sequencing is the most commonly used form of NGS because it is highly accurate, cost-effective, and supported by a wide range of bioinformatic tools. However, obtaining one contiguous genome sequence might be challenging because of repetitive regions in a pathogen’s genome and/or a high percentage of G+C content [56,57,58]. Long reads can improve assembly success by spanning problematic repetitive regions. Nevertheless, long-read sequencing has some limitations, including relatively low accuracy, which introduces errors in sequenced DNA [57,58,59]. A hybrid sequencing approach, combining high-accuracy short read data (Illumina) and long read sequences (Oxford Nanopore Technologies), was applied in this research to produce genome sequences of eight *C. gallinacea* strains (20-339/2, 20-339/3, 20-303/8, 20-291/9, 19-530/1, 19-502/5, 19-473/7, and 19-473/10). The remaining three genome sequences (20-303/10, 19-502/7, and 15-56/1) were obtained based just on second-generation sequencing data (Illumina).

An analysis of the 16S rRNA and 23S rRNA genes of the eleven strains originating from *Gallus gallus* confirmed that they belonged to the *C. gallinacea* species. The ANIb and tetra-nucleotide regression values also supported their species classification. The G+C content of all strains (39.7%) was identical to that of the *C. gallinacea* reference strains 08-1274/3, JX-1, NL F725, and NL G47.

An analysis of the *omp*A gene, as well as MLST, revealed that *C. gallinacea* strains are highly diverse with respect to these loci. Among the eleven strains sequenced, eight novel sequence types have been identified, numbered ST321–ST328. Phylogenetic analysis of the *omp*A gene showed that all strains grouped together with European strains but also with some isolated in Asia.

Comparison of whole genome sequences based on whole-genome SNP analysis clearly showed that *C. gallinacea* strains had conservative genomes. Moreover, an average nucleotide identity comparison of all *Chlamydia* spp. genomes confirmed the low intra-species variability of *C. gallinacea*, with whole-genome sequence identity ranging from 99.34% to even 100%.

The plasticity zone is considered to be a key region of all chlamydial genomes as it is highly variable and associated with pathogenesis [59,60]. The size of this zone ranges by species from 6 kb to 83 kb [61]. Acetyl-CoA carboxylase (*acc*B) and einosine-5′-monophosphate dehydrogenase (*gua*B) are genes determining the boundaries of the PZ in most chlamydiae [59,60]. In *C. gallinacea*, *C. avium*, *C. buteonis*, *C. suis*, and *C. trachomatis*, the lack or truncation of the *gua*AB-*add* operon is common [62]. The analysis of the PZ of the eleven *C. gallinacea* strains researched in the current work (15,791–17,376 bp) revealed that this region is highly conserved, with sequence homology of 90.10–100% with all known *C. gallinacea* genomes. Interestingly, the two clonal strains 20-339/2 and 20-339/3 had an additional CDS assigned as a hypothetical protein of unknown function. In all *C. gallinacea* genomes, MAC/perforin, PLD, and the complete *trp* operon genes were absent; however, the full-length chlamydial cytotoxin (*tox*B) gene could be found. The cytotoxin gene is an important virulence factor as it is related to acute infection and disease [25,63]. In our strains, as well as in the type strain 08-1274/3, this gene was intact, while, in the Chinese strain JX-1 [25], a premature STOP codon was noted. The presence of the cytotoxin gene is characteristic of only some *Chlamydia* spp.: *C. gallinacea*, *C. psittaci*, *C. buteonis*, *C. felis*, *C. caviae*, and *Cand.* *C. ibidis*. (one copy); *Cand. C. pecorum* and *C. suis* (two copies); and *C. muridarum* (three copies). In contrast, in *C. avium*, *C. abortus*, and *C*. *pneumoniae* it was absent [62]. Further investigation is needed to find out which are the virulence features with which the cytotoxin gene correlates.

All known chlamydial taxa, except *C. abortus*, *Cand.* *C. ibidis*, and human-origin *C. pneumoniae* strains [64], carried a highly conserved plasmid which was not integrated into the genome [65]. Chlamydial plasmids are usually 7.5 kbp, organized into eight open reading frames (ORFs), and considered to be related to the virulence of the strain. All of the *C. gallinacea* strains reported here harbored a plasmid with high sequence homology (97.25–100%) to each other and the *C. gallinacea* reference strains 08-1274/3, JX-1, NL F725, and NL G47. Regarding the homology with the plasmid sequences of other *Chlamydia* spp., those of our eleven strains are most closely related to those of *C. avium* 10DC88 (75.96–76.78%) and least related to those of *C. suis* SWA-14, *C. trachomatis* A/HAR-13, and *C. muridarum* Nigg (below 60%). The impact of plasmid presence in *C. gallinacea* strains needs more research.

The T3SS in chlamydial representatives is a system of structural, chaperone, and secreted effector proteins usually arranged into three or four clusters and scattered over the genome [55]. Interestingly, in other bacterial genomes, T3SS structural genes can be found on plasmids or pathogenicity islands along with their effectors [55,66]. In our eleven strains, 75 effectors, chaperones, and structural proteins were determined. The function of most identified T3S effectors is unknown; however, some of them have been elucidated, such as CopN with multiple roles in pathogenesis [67], MrcA promoting chlamydial extrusion [68] or NUE participating in histone methylation [69]. A pair of T3S effector proteins known for their role in chlamydial pathogenesis, TARP and SINC, were found in all eleven strains. The former functions to remodel the host-actin cytoskeleton during the initial stage of infection and facilitate internalization by the host cell [70,71], while the latter is a new T3S protein discovered in *Chlamydia psittaci* which targets the inner nuclear membrane of infected cells and uninfected neighboring cells [72]. The discovery of TARP and SINC in the present research will, however, need to be echoed in more data for it to be finally confirmed that TARP and SINC effector proteins are actually secreted in *C. gallinacea* and for their role in the pathogenicity of this species.

Turning our attention to other putative virulence factors, the chlamydial protease-like activating factor (CPAF) exoenzyme, which was stated to be important in *Chlamydia* virulence [73], was found in all strains presently analyzed, in contrast to the finding of a previously published paper [20], where CPAF was not identified in Dutch *C. gallinacea* representatives NL F725 or NL G47. Moreover, *hct*A and *hct*B, which play principal roles in the conversion of reticulate bodies into elementary bodies, were detected in all *C. gallinacea* strains from this study. Interestingly, these genes were also found in the *C. gallinacea* 08-1274/3 type strain, which is at variance with previously published data, probably due to differences in the annotation tools used [54].

A substantial role in virulence is also played by Inc proteins. They are mainly transported by a T3SS and are well known for their subversion of host cell functions as they decorate the cytosolic face of the inclusion of *Chlamydia* spp. [74]. There is a remarkable divergence in Inc protein content across chlamydial species. To date, seven Inc subtypes (A–G), as well as a newly recognized subtype, *Inc*V, have been described [75]. Taking into consideration differences in nomenclature, it should be mentioned that inclusion membrane proteins are characterized by at least one bi-lobed hydrophobic motif; therefore, the in silico prediction is based on the bi-lobed hydrophobic domains occurring in these proteins [76,77]. In this study, four subtypes were identified by a BLAST search: *Inc*A (two proteins), *Inc*B (one protein), *Inc*C (one protein), and *Inc*V (one protein). Using the DeepTMHMM tool, 20–25 Incs with two transmembrane domains (two *Inc*A and *Inc*C proteins included), 16 with four transmembrane domains (*Inc*V protein included), and 20–21 with six transmembrane domains were predicted. Interestingly, the presence of transmembrane domains in *Inc*B was not confirmed, despite previously published results [25] indicating that there are two transmembrane domains in this protein. This discrepancy was possibly because the analysis referred to was performed on a previous version of DeepTMHMM. A further discrepancy with the literature data is that the number of predicted Incs in *C. gallinacea* strains from this study varies from those noted by Heijne et al. [20], which might be caused by the use of different servers for prediction and by the application of different thresholds. The DeepTMHMM bioinformatic tool used for analysis in this study is currently considered to provide the most complete results [50].

Polymorphic membrane proteins occur in all *Chlamydia* spp. and are regarded as virulence factors [51]. These proteins are highly variable in size, sequence, and number across species [54]. It should be emphasized that the analysis of pmps was demanding. Firstly, the gene annotation was not straightforward because the occurrence of poly-G sequences (mainly in the *pmp*E and *pmp*I genes) generated frameshifts which led to the appearance of premature STOP codons. The issues with the estimation of the correct number of bases in homopolymer regions are well known, especially in short-read technologies; however, they are present in long-read sequencing as well. Moreover, identifying *pmp* genes in multiple contig genomes was additionally challenging. Furthermore, the *pmp* genes and genome sequences available in the GenBank and UniProt databases were often not properly annotated, and pmps nomenclature in databases and publications was inconsistent. Similarly to their detection noted in published research, all the subtypes of the *pmp* genes (A, B, D, E/F, G/I, and H) were detected in our strains [20,25,54]. Taking into consideration the nomenclature variation among chlamydial species, the *pmp*05 gene identified in our *C. gallinacea* strains was assigned to subtype G [25,51,53]. The A, B, D, H, G1, and G2 subtype *pmp* genes seemed to be present and intact in all eleven strains and shared high sequence similarities with each other and with the type strain. In contrast, *pmp*E was not detected in five of the eleven strains, and, also, members of subtypes F and G/I seemed to appear sporadically in different numbers and had a premature STOP codon. However, these observed differences in pmps composition between *C. gallinacea* strains might have been due to sequencing and annotation discrepancies and could not be clearly verified with the bioinformatic tools used in this study. 

Virulence genes are one of the most significant incompletely elucidated aspects of genome analysis in *Chlamydia* spp. Answers to the remaining questions about virulence are not to be fully gained from computational methods based on homology searches, because they have not been experimentally validated [55]. To find out what the function of the gene is, the protein needs to be exhaustively examined. Sequence similarity to genes might offer a good first indication of their function. However, most chlamydial genes are not fully annotated and remain as predictions, although these predictions are often supported by homologs that have been confirmed to be real genes in other strains or species. Furthermore, the majority of genes in *Chlamydia* spp. are hypothetical proteins with unspecified functions [55]. The present study supports the conclusion of Heijne et al. [20] that *C. gallinacea* strains share a high level of sequence identity, with genetic diversity limited to several regions, and possess a reduced set of virulence factors in comparison to *C. psittaci*. In this research, it was confirmed that Polish strains possess virulence genes including cytotoxin, Incs, pmps, histone-like proteins *hct*A and *hct*B, exoenzyme CPAF, T3SS, and the SINC and TARP effectors of T3SS involved in chlamydial pathogenesis. However, further studies are needed to confirm their true involvement in pathogenesis.

## 5. Conclusions

All *C. gallinacea* genomes are closely related, sharing at least 99.46% of genome sequence homology. Regardless of the *C. gallinacea* strain origin, their genomes have a conservative structure considering the plasticity zone, the T3SS (including the SINC and TARP effectors), Incs, pmps, *hct*A and *hct*B, CPAF, and plasmids. However, some regions, such as *omp*A and MLST target genes, are diverse enough to enable high-resolution genotyping and epidemiological tracing. Surprisingly, there was some heterogeneity in strains deriving from the same flock, these differing in the ST, *omp*A genotype, and organization of pmps. Research approaches beyond comparative genomics are needed to clarify the pathogenic potential of *C. gallinacea* and to determine its significance for the poultry industry and for public health.

## Figures and Tables

**Table 1:** Real-time PCR results of selected *C. gallinacea*-positive chicken samples.

No. of Flock	Sample			Real-Time PCR (Ct Value)	
	ID	Type	Origin	*Chlamydiaceae* 23S rRNA	*C. gallinacea*
20-339	20-3392	cloacal swab	chicken	27.95	27.23
	20-339/3	cloacal swab	chicken	29.03	28.13
20-303	20-303/8	cloacal swab	chicken	28.31	26.35
	20-303/10	cloacal swab	chicken	25.88	23.59
20-291	20-291/9	cloacal swab	chicken	29.61	27.73
19-530	19-530/1	cloacal swab	chicken	31.16	30.35
19-502	19-502/5	cloacal swab	chicken	28.19	26.98
	19-502/7	cloacal swab	chicken	26.61	25.21
19-473	19-473/7	cloacal swab	chicken	33.04	31.68
	19-473/10	cloacal swab	chicken	27.99	26.94
15-56	15-56/1	cloacal swab	chicken	23.26	21.80

**Table 2:** Multi-locus sequence typing profiles of *C. gallinacea* field strains.

Sample	*gat*A	*opp*A	*hfl*X	*gid*A	*eno*A	*hem*N	*fum*C	ST
20-339/2	44	81	38	45	36	29	28	321
20-339/3	44	81	38	45	36	29	28	321
20-303/8	44	36	38	45	110	83	28	322
20-303/10	44	36	38	45	110	83	28	322
20-291/9	44	36	40	45	36	29	28	323
19-530/1	44	36	103	45	36	29	28	324
19-502/5	44	36	102	45	36	29	28	325
19-502/7	44	88	38	45	36	84	28	326
19-473/7	44	36	38	45	36	29	89	327
19-473/10	44	36	38	45	36	29	89	327
15-56/1	44	36	102	45	111	29	28	328

## Data Availability

The genome sequences of the strains 20-339/2, 20-339/3, 20-303/8, 20-291/9, 19-530/1, 19-502/5, 19-473/7, 19-473/10, 20-303/10, 19-502/7, 15-56/1 obtained in this study have been deposited in ENA/GenBank/DDBJ under the accession numbers OX332823–OX332824, OX332761–OX332762, OX332828–OX332829, CAMPGD010000001–CAMPGD010000028, CAMPFX010000001–CAMPFX010000011, OX332641–OX332642, CAMPGA010000001–CAMPGA010000017, CAMPFW010000001–CAMPFW010000013, CAMPFY010000001–CAMPFY010000016, CAMPFZ010000001–CAMPFZ010000007, and CAMPGB010000001–CAMPGB010000023, respectively. The respective raw reads of the strains have been deposited under accession numbers ERR10109138, ERR10109123, ERR10109139, ERR10109124, ERR10109137, ERR10109125, ERR10109126, ERR10109136, ERR10109127, ERR10109140, ERR10109128, ERR10109135, ERR10109129, ERR10109130, ERR10109141, ERR10109131, ERR10109134, ERR10109132, and ERR10109133. All data related to WGS of these *C. gallinacea* strains are included as a part of BioProject PRJEB55472.

## Supplementary Materials

The following supporting information can be downloaded at: https://www.mdpi.com/article/10.3390/pathogens12070891/s1, Table S1. Basic genomic parameters of *C. gallinacea* isolates. Table S2. Accession numbers of *C. gallinacea* strains analyzed in this study. Table S3. Pairwise comparison of selected representatives of *Chlamydia* spp. including *C. gallinacea* strains. The lower triangle represents pairwise sequence identity values for the tetra-nucleotide signature correlation index, while the upper triangle shows pairwise sequence identity values for the average nucleotide identity. Table S4. Nucleotide identity values (%) of *omp*A sequences of selected *Chlamydia* spp. Distance matrices based on multiple sequence alignment were used for calculation of nucleotide sequence identity values (Geneious Pro 8.0 software; Biomatters, Auckland, New Zealand). Table S5. The number of single nucleotide polymorphisms of *C. gallinacea* representatives. Table S6. Nucleotide identity values (%) of plasticity zone sequences of selected representative strains of *Chlamydia* spp. including *C. gallinacea* strains. Distance matrices based on multiple sequence alignment were used for calculation of nucleotide sequence identity values (Geneious Pro 8.0 software; Biomatters, Auckland, New Zealand). Table S7. Nucleotide identity values (%) of plasmid sequences of selected *Chlamydia* spp. Distance matrices based on multiple sequence alignment were used for calculation of nucleotide sequence identity values (Geneious Pro 8.0 software; Biomatters, Auckland, New Zealand). Table S8. Putative virulence factors and effector delivery system in *C. gallinacea* strains from this study based on Virulence Factors Database. All type III secretion (T3S) proteins are colored yellow (blue font: effectors, black font: remaining type III secretion system (T3SS) proteins). Table S9. Amino acid identity values (%) of translocated actin recruiting phosphoprotein sequences of *C. gallinacea* representatives. Distance matrices based on multiple sequence alignment were used for calculation of amino acid sequence identity values (Geneious Pro 8.0 software; Biomatters, Auckland, New Zealand). Table S10. Amino acid identity values (%) of secreted inner nuclear membrane-associated *Chlamydia* protein sequences of *C. gallinacea* representatives and the *C. psittaci* 6BC type strain. Distance matrices based on multiple sequence alignment were used for calculation of amino acid sequence identity values (Geneious Pro 8.0 software; Biomatters, Auckland, New Zealand). Table S11. Amino acid identity values (%) of *Inc*A, *Inc*B, *Inc*C, and *Inc*V sequences of *C. gallinacea* representatives. Distance matrices based on multiple sequence alignment were used for calculation of amino acid sequence identity values (Geneious Pro 8.0 software; Biomatters, Auckland, New Zealand). Table S12. Polymorphic membrane proteins (pmps) in *C. gallinacea* strains from this study based on BLAST hits and Virulence Factors Database. Figure S1. Phylogenetic tree of concatenated sequences of seven multi-locus sequence typing housekeeping genes (*eno*A, *fum*C, *gat*A, *gid*A, *hem*N, *hlf*X, and *opp*A) for *C. gallinacea* strains from this survey (in bold), for *C. gallinacea* representatives available in PubMLST database and for *C. avium* 10DC88. Sequence types (ST) are given in brackets for all *C. gallinacea* strains. Figure S2. EasyFig plot of phylogenetic comparison of *C. gallinacea* and *Chlamydia* spp. plasmids based on gene arrangement. Colored arrows represent coding regions, whilst gray shading represents tBLASTx matches. Support values are presented on the branches.
