# Supplementary material for: Whole Genome Sequencing and Comparative Genomic Analysis of Chlamydia gallinacea Field Strains Isolated from Poultry in Poland
Source: Pathogens. 2023 Jun 29;12(7):891. doi: 10.3390/pathogens12070891 (PMC10384503; doi:10.3390/pathogens12070891)
Supplement: Supplementary file 1 [file pathogens-12-00891-s001.zip › Supplementary Table S1.pdf]

Supplementary Table S1. Basic genomic parameters of *C. gallinacea* isolates.

	Feature	20-339/2	20-339/3	20-303/8	20-303/10	20-291/9	19-530/1	19-502/5	19-502/7	19-473/7	19-473/10	15-56/1	*C. gallinacea* 08-1274/3	*C. gallinacea* JX-1	*C. gallinacea* NL F725	*C. gallinacea* NL G47
Basic data	Host	chicken	chicken	chicken	chicken	chicken	chicken	chicken	chicken	chicken	chicken	chicken	chicken	chicken	chicken	chicken
	Country	Poland	Poland	Poland	Poland	Poland	Poland	Poland	Poland	Poland	Poland	Poland	France	China	The Netherlands	The Netherlands
	Year	2020	2020	2020	2020	2020	2019	2019	2019	2019	2019	2015	2008	2014	2017	2017
	Type of sample	cloacal swab	cloacal swab	cloacal swab	cloacal swab	cloacal swab	cloacal swab	cloacal swab	cloacal swab	cloacal swab	cloacal swab	cloacal swab	cloacal swab	cloacal swab	cloacal swab	cloacal swab
Genome	Circular	+	+	+	-	-	+	-	-	-	-	-	+	+	-	-
	Length	1,068,768	1,068,772	1,066,985	1,066,055	1,066,732	1,067,729	1,064,556	1,070,982	1,064,106	1,068,547	1,065,443	1,067,202	1,067,014	1,064,516	1,066,007
	Coverage	320	480	580	844	277	321	50	1520	38	186	340	1949	100	181	366
Chromosome	Length (bp)	1,061,276	1,061,280	1,059,493	1,056,936	1,059,240	1,060,237	1,057,064	1,063,015	1,056,614	1,061,055	1,056,773	1,059,583	1,059,522	1,057,024	1,058,515
	No of contigs	1	1	1	27	10	1	16	12	15	6	22	1	1	19	11
	N_{50}	1,061,276	1,061,280	1,059,493	339,502	1,054,768	1,060,237	565,275	339,536	565,359	1,056,858	359,465	1,059,583	1,059,522	96,259	114,660
	L_{50}	NA	NA	NA	2	1	NA	1	2	1	1	2	NA	NA	4	2
	G+C content (%)	37.9	37.9	37.9	37.9	37.9	37.9	37.9	37.9	37.9	37.9	37.9	37.9	37.9	37.9	37.9
	No. of CDS	910	910	909	899	907	904	899	904	907	910	900	909	918	903	907
	rRNA genes	3	3	3	3	3	3	3	3	3	3	3	3	3	3	3
	tRNA	39	39	39	39	39	39	39	39	39	39	39	39	39	39	39
	tnRNA	1	1	1	1	1	1	1	1	1	1	1	1	1	1	1
	Hypothetical proteins	359	359	357	356	358	355	353	357	362	357	357	354	359	354	359
Plasmid	Length (bp)	7492	7492	7492	7569	7492	7492	7492	7569	7492	7492	7569	7619	7492	7492	7492
	G+C content (%)	31.7	31.7	31.7	31.6	31.8	31.8	31.8	31.7	31.7	31.7	31.6	31.7	31.8	31.8	31.8
	No. of ORFs	8	8	8	8	8	8	8	8	8	8	8	8	8	8	8
