# Supplementary material for: Whole Genome Sequencing and Comparative Genomic Analysis of Chlamydia gallinacea Field Strains Isolated from Poultry in Poland
Source: Pathogens. 2023 Jun 29;12(7):891. doi: 10.3390/pathogens12070891 (PMC10384503; doi:10.3390/pathogens12070891)
Supplement: Supplementary file 1 [file pathogens-12-00891-s001.zip › Supplementary Figure S1.pdf]

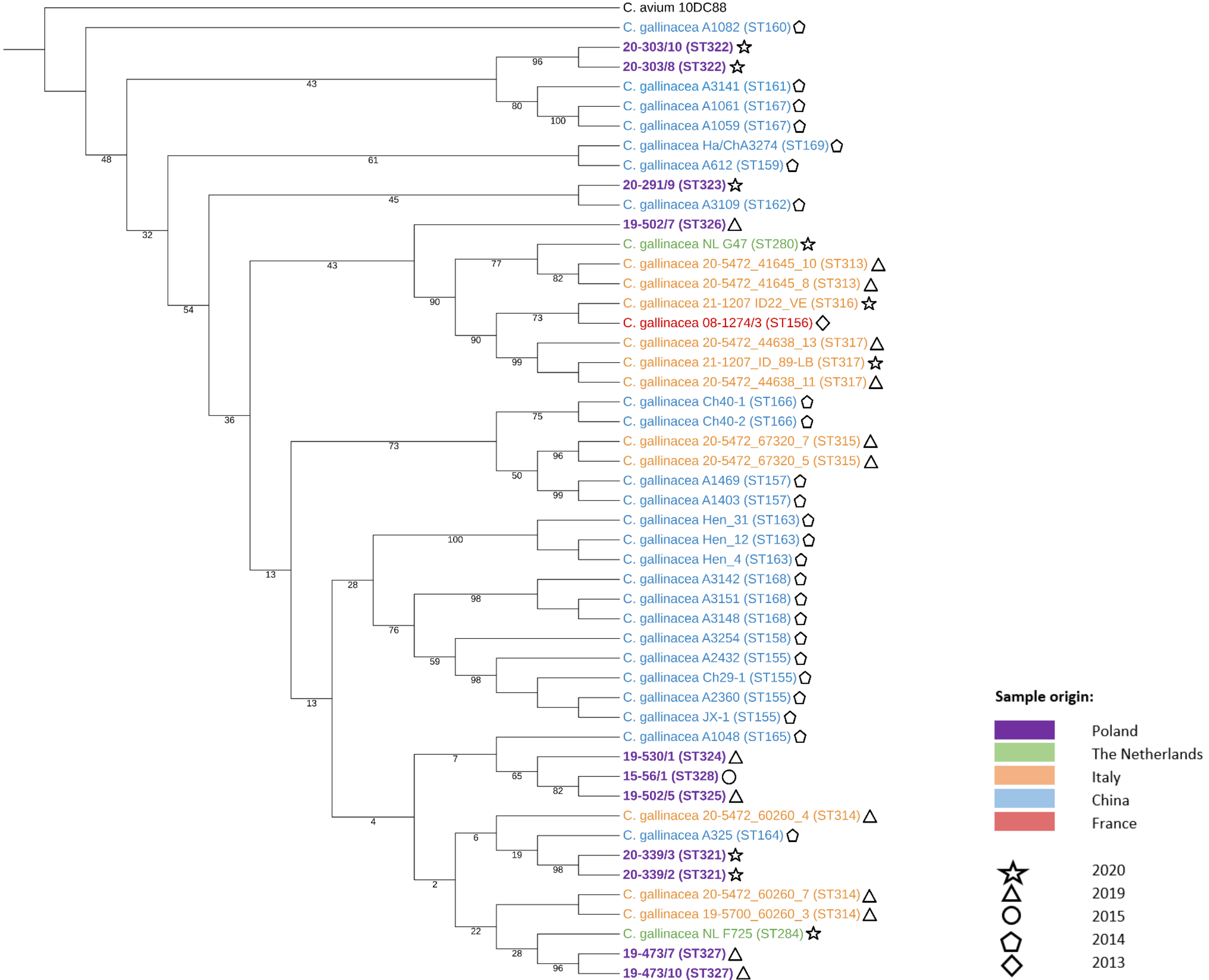
C. avium 10DC88
C. gallinacea A1082 (ST160)
20-303/10 (ST322)
20-303/8 (ST322)
C. gallinacea A3141 (ST161)
C. gallinacea A1061 (ST167)
C. gallinacea A1059 (ST167)
C. gallinacea Ha/ChA3274 (ST169)
C. gallinacea A612 (ST159)
20-291/9 (ST323)
C. gallinacea A3109 (ST162)
19-502/7 (ST326)
C. gallinacea NL G47 (ST280)
C. gallinacea 20-5472_41645_10 (ST313)
C. gallinacea 20-5472_41645_8 (ST313)
C. gallinacea 21-1207 ID22_VE (ST316)
C. gallinacea 08-1274/3 (ST156)
C. gallinacea 20-5472_44638_13 (ST317)
C. gallinacea 21-1207_ID_89-LB (ST317)
C. gallinacea 20-5472_44638_11 (ST317)
C. gallinacea Ch40-1 (ST166)
C. gallinacea Ch40-2 (ST166)
C. gallinacea 20-5472_67320_7 (ST315)
C. gallinacea 20-5472_67320_5 (ST315)
C. gallinacea A1469 (ST157)
C. gallinacea A1403 (ST157)
C. gallinacea Hen_31 (ST163)
C. gallinacea Hen_12 (ST163)
C. gallinacea Hen_4 (ST163)
C. gallinacea A3142 (ST168)
C. gallinacea A3151 (ST168)
C. gallinacea A3148 (ST168)
C. gallinacea A3254 (ST158)
C. gallinacea A2432 (ST155)
C. gallinacea Ch29-1 (ST155)
C. gallinacea A2360 (ST155)
C. gallinacea JX-1 (ST155)
C. gallinacea A1048 (ST165)
19-530/1 (ST324)
15-56/1 (ST328)
19-502/5 (ST325)
C. gallinacea 20-5472_60260_4 (ST314)
C. gallinacea A325 (ST164)
20-339/3 (ST321)
20-339/2 (ST321)
C. gallinacea 20-5472_60260_7 (ST314)
C. gallinacea 19-5700_60260_3 (ST314)
C. gallinacea NL F725 (ST284)
19-473/7 (ST327)
19-473/10 (ST327)
Sample origin:
Poland
The Netherlands
Italy
China
France
2020
2019
2015
2014
2013
