# Supplementary material for: Whole Genome Sequencing and Comparative Genomic Analysis of Chlamydia gallinacea Field Strains Isolated from Poultry in Poland
Source: Pathogens. 2023 Jun 29;12(7):891. doi: 10.3390/pathogens12070891 (PMC10384503; doi:10.3390/pathogens12070891)
Supplement: Supplementary file 1 [file pathogens-12-00891-s001.zip › Supplementary Table S2.pdf]

Supplementary Table S2. Accession numbers of *C. gallinacea* strains analyzed in this study.

No.	ASSEMBLY_NAME	STUDY_ID	SAMPLE_ID	RAW_DATA	ASSEMBLY_ACC	CONTIG_ACC	CHROMOSOME_ACC
**1.**	*Chlamydia gallinacea* strain 20-339/2	PRJEB55472	ERS12768539	ERR10109123 (Illumina MiSeq)<br>ERR10109138 (MinION)	GCA_946862435	-	OX332823-OX332824
**2.**	*Chlamydia gallinacea* strain 20-339/3	PRJEB55472	ERS12768540	ERR10109124 (Illumina MiSeq)<br>ERR10109139 (MinION)	GCA_946861635	-	OX332761-OX332762
**3.**	*Chlamydia gallinacea* strain 20-303/8	PRJEB55472	ERS12768541	ERR10109125 (Illumina MiSeq)<br>ERR10109137 (MinION)	GCA_946862465	-	OX332828-OX332829
**4.**	*Chlamydia gallinacea* strain 20-303/10	PRJEB55472	ERS12768542	ERR10109126 (Illumina NovaSeq)	GCA_946868125	CAMPGD010000001-CAMPGD010000028	-
**5.**	*Chlamydia gallinacea* strain 20-291/9	PRJEB55472	ERS12768543	ERR10109127 (Illumina MiSeq)<br>ERR10109136 (MinION)	GCA_946859895	CAMPFX010000001-CAMPFX010000011	-
**6.**	*Chlamydia gallinacea* strain 19-530/1	PRJEB55472	ERS12768544	ERR10109128 (Illumina MiSeq)<br>ERR10109140 (MinION)	GCA_946859875	-	OX332641-OX332642
**7.**	*Chlamydia gallinacea* strain 19-502/5	PRJEB55472	ERS12768545	ERR10109129 (Illumina MiSeq)<br>ERR10109135 (MinION)	GCA_946863755	CAMPGA010000001-CAMPGA010000017	-
**8.**	*Chlamydia gallinacea* strain 19-502/7	PRJEB55472	ERS12768546	ERR10109130 (Illumina NovaSeq)	GCA_946859795	CAMPFW010000001-CAMPFW010000013	-
**9.**	*Chlamydia gallinacea* strain 19-473/7	PRJEB55472	ERS12768547	ERR10109131 (Illumina MiSeq)<br>ERR10109141 (MinION)	GCA_946859935	CAMPFY010000001-CAMPFY010000016	-
**10.**	*Chlamydia gallinacea* strain 19-473/10	PRJEB55472	ERS12768548	ERR10109132 (Illumina MiSeq)<br>ERR10109134 (MinION)	GCA_946863835	CAMPFZ010000001-CAMPFZ010000007	-
**11.**	*Chlamydia gallinacea* strain 15-56/1	PRJEB55472	ERS12768549	ERR10109133 (Illumina NovaSeq)	GCA_946864325	CAMPGB010000001-CAMPGB010000023	-
