# Supplementary figures and images for: Whole Genome Sequencing and Comparative Genomic Analysis of Chlamydia gallinacea Field Strains Isolated from Poultry in Poland
Source: Pathogens. 2023 Jun 29;12(7):891. doi: 10.3390/pathogens12070891 (PMC10384503; doi:10.3390/pathogens12070891)

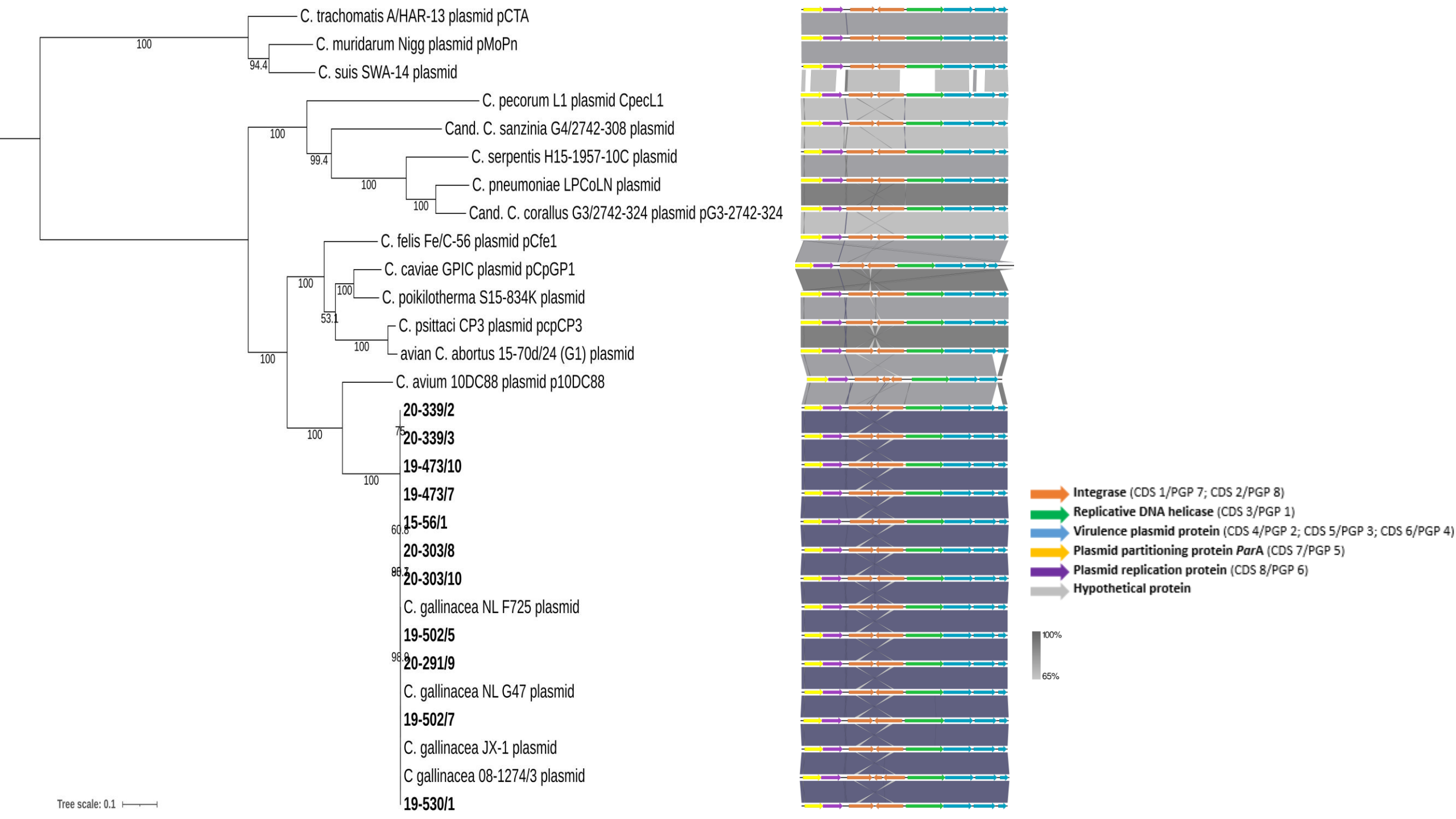

Supplement: Supplementary file 1 [file pathogens-12-00891-s001.zip › Supplementary Figure S2.pdf]
